# Knowledge and Attitudes of Healthcare Workers Toward the Re‐Emergence of Human Monkeypox Virus Infection in Public Health Facilities in the Central Ethiopia Region: An Online Cross‐Sectional Study

**DOI:** 10.1002/hsr2.72854

**Published:** 2026-07-30

**Authors:** Yilma Markos Larebo, Zerfework Debebe Argago, Markos Selamu Jifar, Abebe Alemu Anshebo, Sujit Kumar Behera, Natarajan Gopalan

**Affiliations:** ^1^ Department of Public Health Wachemo University Hossana Ethiopia; ^2^ Department of Midwifery Wachemo University Hossana Ethiopia; ^3^ Department of Epidemiology and Public Health, School of Life Science Central University of Tamil Nadu Thiruvarur India

**Keywords:** attitude, Central Ethiopia, healthcare worker, knowledge, monkeypox virus

## Abstract

**Background and Aims:**

Human monkeypox (mpox) is a re‐emerging zoonotic viral disease in Africa. Despite recent reports in Ethiopia, no studies have assessed healthcare workers' (HCWs) knowledge and attitudes in the Central Ethiopia Region. This study aimed to determine HCWs knowledge and attitudes toward the re‐emergence of mpox in public health facilities.

**Methods:**

From June 16 to 28, 2025, 419 HCWs were chosen at random for an online cross‐sectional study. The data were gathered using a self‐administered questionnaire distributed through Google Forms. We used SPSS version 26 for the statistical analysis. Multivariable logistic regression was used to identify factors associated with knowledge and attitudes. Adjusted odds ratios (AORs) with 95% confidence intervals (CIs) were used to determine statistical significance.

**Results:**

Of the 419 participants, 46.8% (95% CI: 42.0–51.7) had good knowledge, while 43.4% (95% CI: 39.1–48.0) showed positive attitudes. Good knowledge were significantly positively associated with a master's degree or higher (AOR = 3.46; 95% CI: 1.60–7.47), health officer (AOR = 7.00; 95% CI: 3.20–15.32) and pharmacist (AOR = 2.68; 95% CI: 1.17–6.13), low (AOR = 5.15; 95% CI: 2.02–13.10) and medium monthly income (AOR = 2.35; 95% CI: 1.32–4.20), and positive attitude (AOR = 2.11; 95% CI: 1.25–3.59), in contrast significantly negatively associated with work experience (5–10 years) (AOR = 0.13; 95% CI: 0.07–0.24). Positive attitudes were significantly positively associated with medical doctor (AOR = 4.78; 95% CI: 2.39–9.55), and information about mpox during medical education (AOR = 3.29; 95% CI: 1.91–5.67), in contrast significantly negatively associated with female sex (AOR = 0.18; 95% CI: 0.08–0.41), age ≥ 32 years (AOR = 0.45; 95% CI: 0.28–0.73), and lack of current information access (AOR = 0.47; 95% CI: 0.26–0.88).

**Conclusion:**

HCWs had low levels of knowledge and positive attitudes toward mpox. To get better at being ready for and responding to mpox in Ethiopia, it is important to strengthen continuing professional education, target training, and raise awareness.

## 1 Background

Monkeypox is a re‐emerging zoonotic infectious disease caused by the monkeypox virus, a double‐stranded DNA virus belonging to the Poxviridae family [1, 2, 3, 4, 5, 6]. In recent years, mpox has gained global attention due to its expanding geographic distribution and increasing number of cases worldwide, leading to its declaration as a public health emergency of international concern by the World Health Organization (WHO) in 2022 [2, 4, 6, 7, 8, 9, 10].

Historically, mpox was first identified in 1958 in Denmark [10, 11, 12, 13], with the first human case reported in 1970 in the Democratic Republic of the Congo [1, 3, 5, 7, 11, 12, 14, 15]. The disease is now common in many countries in West and Central Africa and has spread to more than 120 countries around the world. As of 2024, there have been over 100,000 confirmed cases [6, 16]. Recent outbreaks also affected several African countries, showing that the virus is still a public health threat [6, 17].

Mpox is primarily transmitted through close contact with infected individuals, animals, or contaminated materials, and less commonly through respiratory droplets [3, 4, 5, 8, 11]. The disease usually shows up with a fever, swollen lymph nodes, and a rash that looks like the rash from other infections, like smallpox, which makes it hard for medical professionals to identify what it is [1, 3, 5, 16]. Mpox is usually self‐limiting, but it can cause serious problems in some cases, especially in people who are already weak [3, 16].

The recent rise in mpox cases shows how important it is to prevent the disease, find it early, and manage cases correctly. Healthcare workers are essential to these efforts; nonetheless, deficiencies in knowledge and awareness have been recognized as major obstacles to disease control [12, 18]. The first confirmed mpox case in Ethiopia was reported in 2025, and more cases have since been reported, showing that the disease could spread further [19].

Even though there have been recent mpox reports in Ethiopia, there isn't much information about healthcare workers' knowledge and attitudes, especially in the Central Ethiopia Region. Understanding these factors is essential for informing targeted interventions, improving preparedness, and strengthening response strategies.

Therefore, this study aims to determine the knowledge and attitudes of healthcare workers toward the re‐emergence of human monkeypox virus infection in public health facilities in the Central Ethiopia Region.

## 2 Methods and Materials

### 2.1 Study Design, Period, and Setting

An institution‐based online cross‐sectional study was conducted among healthcare workers in the central Ethiopia region from June 16 to 28, 2025. The central Ethiopian region is 232 km from Addis Ababa, the capital city of Ethiopia. The estimated total population of the area is 6,430,235, consisting of 3,186,824 men (49.56%) and 3,243,411 women (50.44%) [20]. The region comprises seven zones and three special districts, with 1656 public and private health facilities (two comprehensive specialized hospitals, five general hospitals [GHs], 21 primary hospitals, 228 health centers, 1067 health posts, and 333 private clinics). The region employs 13,995 healthcare workers [21].

Ethiopia's healthcare system is made up of three levels, with a strong focus on preventive care. Health posts (3000–5000 people), health centers (15,000–25,000 people), and primary hospitals (60,000–100,000 people) are all part of the first level. Health centers serve as the first point of contact for approximately 40,000 people in urban areas. The secondary level includes general hospitals serving 1–1.5 million people, while the tertiary level consists of specialized referral hospitals serving 3.5–5 million people [22]. This system is managed hierarchically by the Federal Ministry of Health (FMOH), Regional Health Bureaus (RHBs), Zonal Health Departments (ZHDs), and Woreda Health Offices, with primary healthcare at the kebele level delivered mainly by Health Extension Workers (HEWs) [23].

Public health facilities in the Central Ethiopia Region, where this study was conducted, provide a range of preventive and curative services, including maternal and child health, immunizations, outpatient and inpatient care, and laboratory diagnostics [24]. These facilities are staffed by medical doctors, health officers, nurses, midwives, laboratory professionals, and pharmacists.

Ethiopia has a low health workforce density of 0.96 per 1000 people, far below the African average (2.2/1000), and the WHO minimum threshold of 4.45/1000 is needed to achieve universal health coverage (UHC). Ethiopia's total health workforce numbered approximately 219,500, of which around 150,500 (68%) were core health professionals (medical doctors, health officers, nurses, midwives, pharmacists, environmental health workers, laboratory technologists, etc.) for a population exceeding 132 million [25, 26].

Medical doctors undergo 6 years of training, and health officers complete a 4‐year Bachelor of Science (BSc) program. At the same time, nurses and midwives follow 3‐year diploma or 4‐year degree programs, while pharmacists complete a 5‐year BSc in pharmacy with extensive pharmaceutical training. In contrast, environmental health workers are trained through 3‐year diplomas or 4‐year BSc programs focusing on sanitation, water safety, and disease prevention. Training institutions produce only about 10,000 graduates annually, insufficient to meet the country's growing demand [26].

### 2.2 Sample Size Determination

The sample size was estimated using the Cochrane formula for a single population proportion with the following consideration: *p* = 38.5% (where *p* represents the prevalence study conducted among healthcare workers to assess their knowledge in Injibara General Hospital, Northwest Ethiopia, to maximize the sample size) [1], a critical value (*Zα*/2) of 1.96 at a 95% confidence level, and a desired margin of error (*d*) of 5%, as shown below.

*n* = (*Zα*/2)^2^ × *P* (1 − *P*)/*d* ^2^

*n* = (1.96)^2^ × 0.385 (1 − 0.385)/(0.05)^2^ = 364

Adjusting for a 15% nonresponse rate increased the sample size by 55 participants, yielding a final sample of 419.

### 2.3 Population

The source population consisted of all healthcare workers employed in public health institutions within the region. In contrast, the study population consisted of healthcare workers delivering services at selected public health facilities throughout the region during the data collection period.

### 2.4 Inclusion and Exclusion Criteria

The study focused on healthcare workers who chose to participate and provided their consent. We made sure to respect everyone's decision, so those who either didn't consent or sent incomplete answers were not included in the study.

### 2.5 Sampling Procedure

Healthcare workers were recruited via social media platforms using convenience sampling, including Facebook, Messenger, WhatsApp, LinkedIn, Telegram, Instagram, Twitter, and email. Participation was voluntary, and no incentives were provided. Informed consent was obtained electronically by asking participants to provide it before the survey began. Only fully completed questionnaires were included in the sample, yielding a total of 419 participants.

### 2.6 Operational Definition

Healthcare Workers: This term includes all members of the healthcare team, such as doctors, nurses, midwives, pharmacists, and laboratory professionals, who are directly involved in providing medical care and services to patients. Their roles are essential to delivering quality healthcare [1, 27].

Good Knowledge: If the healthcare worker answers above the mean value of the 37 items of knowledge score question. Poor Knowledge: If the healthcare worker answers below or equal to the mean value of the 37 items of knowledge score question [1, 28, 29]. Positive Attitude: If the healthcare worker answers above the mean value of the 27 items of attitude score question. Negative Attitude: If the healthcare worker answers below or equal to the mean value of the 27 items of attitude score question [1, 28, 29].

Monthly income was categorized into three groups based on Ethiopian Birr (ETB) and its approximate United States Dollar (USD) equivalent as follows: (1) Low income: < 7424 ETB (≈ < 53.88 USD); (2) medium income: 7424–10,129 ETB (≈ 53.88–73.51 USD), and high income: ≥ 10,130 ETB (≈ ≥ 73.51 USD) [1, 27, 30].

### 2.7 Data Collection and Quality Assurance Procedures

Data were collected through an anonymous online survey shared on platforms such as Facebook, Messenger, WhatsApp, LinkedIn, Telegram, Instagram, Twitter, and email. To encourage participation, two reminder messages were sent after the initial invitation. The survey included an introductory page that provided information about the principal investigators, study objectives, and benefits of participation. To ensure confidentiality, no personal identifiers were collected, and access to the data was limited to the principal investigator. The questionnaire took approximately 15–20 min to complete.

The survey tool consisted of three parts: (1) Sociodemographic characteristics, including gender, age, residence, marital status, religion, education level, work experience, profession type, facility level, monthly income, previous training on mpox virus infection, participation in national/international conferences, and access to mpox‐related information; (2) Knowledge, comprising 38 items with response options of “Yes,” “No,” or “I don't know.” Correct answers were scored 1, and incorrect or uncertain responses were scored 0; and (3) Attitude, including 28 items measured on a 5‐point Likert scale (*strongly agree* to *strongly disagree*), which were dichotomized into positive attitudes, which were scored 1, and negative attitudes, which were scored 0.

The questionnaire was initially developed in English, translated into Amharic for consistency, and then back‐translated into English to ensure consistency. The survey was based on validated studies [1, 3, 5, 7, 11, 12, 15, 28, 31, 32, 33, 34]. A pretest with 5% of the target population (*n* = 21) from private health facilities demonstrated clarity and consistency, achieving a Cronbach's alpha of 0.86 and confirming content validity through expert review.

The survey is accessible at: https://forms.gle/g8K2iUMsVgVhZjJ66 (accessed on June 12, 2025). To ensure data completeness, responses to key variables were required before submission, resulting in no missing data for the main variables analyzed, while incomplete responses were excluded before analysis.

### 2.8 Transparency and Rigor

This study was conducted following the STROBE Statement for cross‐sectional studies, ensuring adherence to established standards [35]. Relevant methodological details, including study design, sampling procedures, and statistical analyses, have been clearly outlined to promote transparency.

Data quality was maintained through standardized collection procedures, pretesting of the questionnaire, and internal consistency assessment via Cronbach's alpha. The data set analyzed in this study is available as a Supporting Information file.

### 2.9 Data Processing and Analysis

All data analysis was performed using the Statistical Package for the Social Sciences (SPSS) version 26.0 (IBM Corp., Armonk, NY, USA) [36]. Descriptive statistics summarized the study variables: frequencies, percentages, means, and standard deviations (SDs). Results were presented in tables, graphs, and charts as appropriate. Bivariate logistic regression was performed to examine associations between sociodemographic characteristics and scores on knowledge and attitudes of HCWs regarding mpox virus infection. The multivariable logistic regression model included variables with a *p* value < 0.25 in the bivariable analysis.

Multivariable logistic regression analysis was conducted to identify independent predictors of knowledge and attitudes among HCWs regarding mpox virus infection. Adjusted odds ratios (AORs) with 95% confidence intervals (CIs) were reported after adjustment in the multivariable logistic regression analysis, and statistical significance was set at a *p* value < 0.05. The goodness‐of‐fit of the final model was assessed using the Hosmer–Lemeshow test (*p* = 0.24), indicating good model fit. Multicollinearity among independent variables was evaluated using the variance inflation factor (VIF), with a cutoff value of (VIF = 3.2), suggesting no significant multicollinearity.

### 2.10 Ethical Consideration

Ethical approval was granted by the Research Ethics Committee of the Wachemo University College of Medicine and Health Sciences Institutional Review Board (IRB), with Ethical Approval protocol number WCU‐IRB 0322/2025, dated June 6, 2025. This research followed the principles outlined in the Declaration of Helsinki [37]. The confidentiality and anonymity of the participants' responses were also guaranteed. Informed consent was obtained electronically through a written online consent form, which each participant reviewed and confirmed before taking part in the study. Only those who provided consent were enrolled. No minors were included in this study; therefore, parental or guardian consent was not required.

## 3 Results

### 3.1 Sociodemographic Characteristics of the Study Respondents

The survey gathered 419 responses, achieving a 100% completion rate. The majority of participants were male (359, 85.7%) and aged 32 years or younger (232, 55.4%). A significant proportion (307, 73.3%) resided in urban areas. Regarding personal demographics, 290 (69.2%) were married, and 248 (59.2%) identified as Protestant. Over half held a master's degree or higher (236, 56.3%). The mean age was 32 years (SD ± 5.96), with nurses and midwives constituting the largest professional group at 151 (36%).

**Figure 1:**
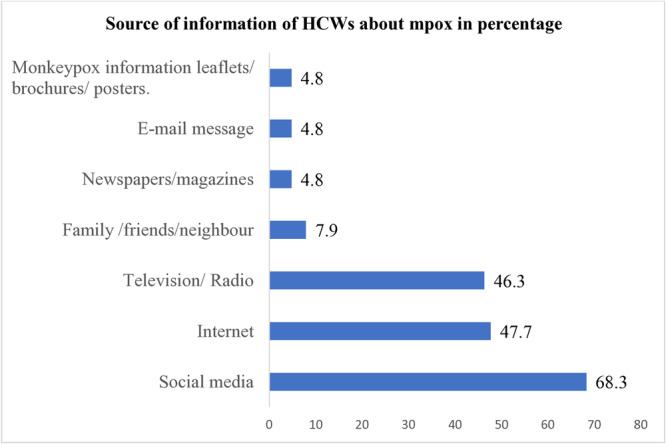
Source of information among healthcare workers regarding monkeypox virus infection in the Central Ethiopia region, 2025 (*n* = 419). HCWs, healthcare workers; mpox, monkeypox.

About 180 (43%) HCWs had 10 or more years of work experience, 148 (35.3%) worked in referral or specialized hospitals, and 195 (46.5%) had higher monthly income. Only 46 (11%) HCWs had received mpox training, 6 (1.4%) had attended national conferences, and none had participated in international conferences. While most participants (296, 70.6%) had heard of mpox, only 139 (33.2%) reported receiving information about mpox infection during their medical education. Overall, 286 (68.3%) HCWs had information about mpox infection. The primary sources of information were social media (286, 68.3%), the Internet (200, 47.7%), Television (TV) or radio (194, 46.3%), and friends or family (33, 7.9%) (Figure 1 and Table 1).

**Table 1:** Sociodemographic‐related characteristics of the healthcare workers toward human monkeypox virus infection in public health facilities in the central Ethiopia region, 2025 (*n* = 419).

Variables	Categories	*n* (%)
Gender	Male	359 (85.7)
	Female	60 (14.3)
Age in years	< 32	232 (55.4)
	≥ 32	187 (44.6)
Residence	Urban	307 (73.3)
	Rural	112 (26.7)
Marital status	Single	129 (30.8)
	Married	290 (69.2)
Religion	Orthodox	92 (22.0)
	Muslim	72 (17.2)
	Protestant	248 (59.2)
	Catholic	7 (1.7)
Educational level	Diploma	146 (34.8)
	Bachelor degree	136 (32.5)
	Master's degree and above	137 (32.7)
Work experience (in years)	≤ 5	107 (25.5)
	5–10	132 (31.5)
	≥ 10	180 (43.0)
Profession type	Laboratory technician/technologist	42 (10.0)
	Nurse/Midwife	151 (36.0)
	Health Officer	72 (17.2)
	Pharmacist	40 (9.5)
	Environmental health	24 (5.7)
	Medical doctor (general practitioner or specialist)	67 (16.0)
	Others	23 (5.5)
Level of the health facility	Health post/health center	130 (31.0)
	Primary hospital/general hospital	141 (33.7)
	Referral/specialized hospital	148 (35.3)
Monthly income	< 7424 ETB (≈ < 53.88 USD) (low income)	48 (11.5)
	7424–10,129 ETB (≈ 53.88–73.51 USD) (medium income)	176 (42.0)
	≥ 10,130ETB (≈ ≥ 73.51 USD) (high income)	195 (46.5)
Trained about the mpox virus infection before	Yes	46 (11.0)
	No	373 (89.0)
Attended a national conference	Yes	6 (1.4)
	No	413 (98.6)
Attended an international conference	Yes	0 (0.0)
	No	419 (100)
Information about the mpox virus infection during your medical education	Yes	139 (33.2)
	No	280 (66.8)
Have you heard about the mpox virus before?	Yes	296 (70.6)
	No	123 (29.4)
Current information about the mpox virus infection	Yes	286 (68.3)
	No	133 (31.7)
Source of information about mpox (more than one possible answer)	Television/radio	194 (46.3)
	Newspapers/magazines	20 (4.8)
	Social media	286 (68.3)
	Internet	200 (47.7)
	Email message	20 (4.8)
	Family/friends/neighbor	33 (7.9)
	mpox information leaflets/brochures/posters	20 (4.8)
First time you heard information about monkeypox	I did not hear about it	34 (8.1)
	Within several days or weeks ago	103 (24.6)
	Within the last month or so	282 (67.3)

*Note:* Where: Others: 13 psychiatrists, 10 health informaticists, ETB: Ethiopian Birr; USD: United States Dollars. Where: HCWs: healthcare workers, mpox: monkeypox.

### 3.2 Knowledge and Attitude of Healthcare Workers Stratified by Profession

When stratified by professional type, the proportion of HCWs with good knowledge of mpox was highest among nurse/midwives (64; 32.7%), followed by health officers (54; 27.6%), medical doctors (general practitioners or specialists) (31; 15.8%), pharmacists (20; 10.2%), laboratory technicians/technologists (11; 5.6%), others (psychiatrist and health informatics) (10; 5.1%), and environmental health (6; 3.1%). Similarly, regarding positive attitudes, nurse/midwives accounted for 52 (28.6%), followed by medical doctors (50; 27.5%), pharmacists (22; 12.1%), health officers (21; 11.5%), laboratory technicians/technologists (17; 9.3%), others (psychiatrist and health informatics) (12; 6.6%), and environmental health (8; 4.4%) (Table 2).

**Table 2:** Knowledge and attitudes of healthcare workers, stratified by professional type, toward monkeypox virus infection in the Central Ethiopia region, 2025 (*n* = 419).

		Knowledge of HCWs toward the mpox virus infection		
Variables	Categories	Poor, *n* (%)	Good, *n* (%)	Total (%)
Profession type	Laboratory technician/technologist	31 (13.9)	11 (5.6)	42 (10.0)
	Nurse/midwife	87 (39.0)	64 (32.7)	151 (36.0)
	Health officer	18 (8.1)	54 (27.6)	72 (17.2)
	Pharmacist	20 (9.0)	20 (10.2)	40 (9.5)
	Environmental health	18 (8.1)	6 (3.1)	24 (5.7)
	Medical doctor (general practitioner or specialist)	36 (16.1)	31 (15.8)	67 (16.0)
	Others	13 (5.8)	10 (5.1)	23 (5.5)
Total (%)		223 (100)	196 (100)	419 (100)

		Attitude of HCWs toward the mpox virus infection		
Variables	Categories	Negative, *n* (%)	Positive, *n* (%)	Total (%)
Profession type	Laboratory technician/technologist	25 (10.5)	17 (9.3)	42 (10.0)
	Nurse/midwife	99 (41.8)	52 (28.6)	151 (36.0)
	Health officer	51 (21.5)	21 (11.5)	72 (17.2)
	Pharmacist	18 (7.6)	22 (12.1)	40 (9.5)
	Environmental health	16 (6.8)	8 (4.4)	24 (5.7)
	Medical doctor (general practitioner or specialist)	17 (7.2)	50 (27.5)	67 (16.0)
	Others	11 (4.6)	12 (6.6)	23 (5.5)
Total (%)		237 (100)	182 (100)	419 (100)

*Note:* Where: Others: 13 psychiatrists, 10 health informaticists.

### 3.3 Knowledge of Healthcare Workers Toward Monkeypox Virus Infection

**Figure 2:**
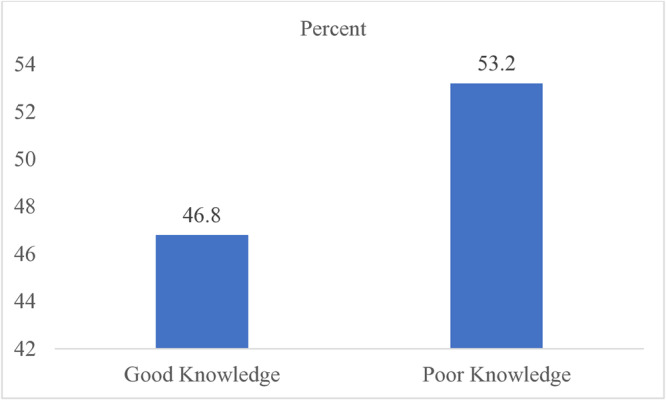
Knowledge‐related characteristics of healthcare workers regarding monkeypox virus infection in the Central Ethiopia region, 2025 (*n* = 419).

Of the 419 study respondents, 196 (46.8%) (95% CI: 42.0–51.7) had good knowledge (Figure 2).

Most HCWs knew that mpox was prevalent in Western and Central Africa (262, 62.5%) but not in Middle Eastern countries (265, 63.2%). Additionally, 157 (37.5%) HCWs were unaware of any confirmed human mpox cases in the United States, Canada, the United Kingdom, or Europe. Most HCWs (385, 91.9%) confirmed the presence of mpox cases in Ethiopia, but 409 (97.6%) did not confirm their presence in the Central Ethiopia Region. Regarding the nature of mpox, most HCWs correctly identified it as a viral illness (392, 93.6%) rather than a bacterial one (399, 95.2%). While the majority, 394 (94%), recognized that mpox could be transmitted through direct skin‐to‐skin contact with an infected person, 365 (87.1%) also correctly stated that it could be transmitted through direct contact with infected animals or the bite of an infected monkey or other animals. However, many HCWs were unaware that mpox shares similar signs and symptoms with chickenpox (226, 53.9%) and smallpox (205, 48.9%). Most participants recognized skin rashes (385, 91.9%), papules (358, 85.4%), vesicles (327, 78.0%), and pustules (305, 72.8%) as signs and symptoms of monkeypox. A significant number also identified flu‐like symptoms (358, 85.4%), lymphadenopathy (307, 73.3%), and diarrhea (153, 36.5%) as associated symptoms. Regarding management, nearly equal proportions of HCWs reported that antiviral drugs (197, 47%) and antibiotics (218, 52%) could treat mpox. Most participants correctly identified paracetamol (319, 76.1%) as part of the management approach. Additionally, 160 (38.2%) and 133 (31.7%) participants believed that individuals infected with smallpox and chickenpox could be vaccinated. However, more than half of the participants incorrectly assumed that a specific vaccine (221, 52.7%) and a specific treatment (339, 80.9%) were available for mpox (Table 3).

**Table 3:** Knowledge‐related characteristics of healthcare workers regarding monkeypox virus infection in the Central Ethiopia region, 2025 (*n* = 419).

Knowledge questions	Categories	*n* (%)
Q1. There is/are confirmed human mpox cases in Ethiopia (Answer: Yes)	Yes	385 (91.9)
	No	34 (8.1)
Q2. There is/are confirmed human mpox cases in the central Ethiopia region (Answer: No)	Yes	9 (2.1)
	No	409 (97.6)
Q3. Mpox is prevalent in Middle Eastern countries (Answer: No)	Yes	154 (36.8)
	No	265 (63.2)
Q4. Mpox is prevalent in Southeast Asian countries (Answer: No)	Yes	158 (37.7)
	No	261 (62.3)
Q5. Mpox is prevalent in Western and Central Africa (Answer: Yes)	Yes	262 (62.5)
	No	157 (37.5)
Q6. There is/are confirmed human mpox cases in the USA, Canada, the UK, or Europe (Answer: Yes)	Yes	223 (53.2)
	No	119 (46.8)
Q7. Since 2022, mpox cases have been reported in more than 130 countries worldwide (Answer: Yes)	Yes	223 (53.2)
	No	196 (46.8)
Q8. Mpox is caused by a newly discovered virus (Answer: No)	Yes	178 (42.5)
	No	241 (57.5)
Q9. Mpox is a re‐emerging disease (Answer: Yes)	Yes	333 (79.5)
	No	86 (20.5)
Q10. Mpox is a viral disease (Answer: Yes)	Yes	392 (93.6)
	No	27 (6.4)
Q11. Mpox is a bacterial disease (Answer: No)	Yes	20 (4.8)
	No	399 (95.2)
Q12. Mpox is transmitted to humans through direct contact with infected animals or bites from an infected monkey or other animal (Answer: Yes).	Yes	365 (87.1)
	No	54 (12.9)
Q13. Mpox can be transmitted through direct skin‐to‐skin contact with an infected person (Answer: Yes)	Yes	394 (94)
	No	25 (6)
Q14. International travel is the main source of imported cases of mpox infection (Answer: Yes)	Yes	298 (71.1)
	No	121 (28.9)
Q15. The mpox virus can cross the placenta from an infected mother to her fetus (Answer: Yes)	Yes	248 (59.2)
	No	171 (40.8)
Q16. Mpox is spread through direct contact with body fluids, sexual activity, kissing, cuddling, contact with sores, or contaminated materials (such as clothing, bedding, or surfaces), as well as through respiratory droplets from coughing or sneezing (Answer: Yes)	Yes	390 (90.7)
	No	39 (9.3)
Q17. Blood‐borne transmission of mpox is possible (Answer: Yes).	Yes	299 (71.4)
	No	120 (28.6)
Q18. Mpox is transmitted through eating food contaminated with the virus (Answer: No)	Yes	261 (62.3)
	No	158 (37.7)
Q19. Mpox can be transmitted by eating insufficiently cooked meat from an infected animal (Answer: Yes).	Yes	280 (66.8)
	No	139 (33.2)
Q20. Mpox and chickenpox have similar signs and symptoms (Answer: Yes).	Yes	193 (46.1)
	No	226 (53.9)
Q21. Mpox and smallpox have similar signs and symptoms (Answer: Yes).	Yes	214 (51.1)
	No	205 (48.9)
Q22. Flu‐like symptoms (fever, chills, cough, runny nose, fatigue, headache, and muscle and backache) are the early signs or symptoms of human mpox (Answer: Yes).	Yes	358 (85.4)
	No	61 (14.6)
Q23. Rashes (an area of irritated or swollen skin) are one of the signs or symptoms of human mpox (Answer: Yes).	Yes	385 (62.3)
	No	158 (37.7)
Q24. Vesicles (small, fluid‐filled sacs) on the skin are one of the signs or symptoms of human mpox (Answer: Yes).	Yes	327 (91.9)
	No	34 (8.1)
Q25. Papules (tiny, raised bumps on the skin) are one of the signs or symptoms of human mpox (Answer: Yes).	Yes	358 (85.4)
	No	61 (14.6)
Q26. Pustules (bulging patches of skin filled with yellowish pus) are one of the signs or symptoms of human monkeypox (Answer: Yes).	Yes	305 (72.8)
	No	114 (27.2)
Q27. Diarrhea is one of the signs or symptoms of human mpox (Answer: No).	Yes	153 (36.5)
	No	266 (63.5)
Q28. Lymphadenopathy (swollen lymph nodes) is one clinical sign that can help differentiate mpox from smallpox (Answer: Yes).	Yes	307 (73.3)
	No	112 (26.7)
Q29. mpox can be prevented by adequately cooking meat from potentially infected animals (Answer: Yes).	Yes	274 (65.4)
	No	145 (34.6)
Q30. Hand sanitizers and face masks prevent mpox (Answer: Yes).	Yes	350 (83.5)
	No	69 (16.5)
Q31. One management option for symptomatic mpox patients is to use paracetamol (Answer: Yes).	Yes	319 (76.1)
	No	100 (23.9)
Q32. Antiviral drugs are required to manage human mpox patients (Answer: Yes).	Yes	197 (47.0)
	No	222 (53.0)
Q33. Antibiotics are required to manage human mpox patients (Answer: No).	Yes	218 (52.0)
	No	201 (48.0)
Q34. There is a specific vaccine for mpox (Answer: Yes).	Yes	198 (47.3)
	No	221 (52.7)
Q35. People who got the smallpox vaccine are immunized against mpox (Answer: No).	Yes	160 (38.2)
	No	259 (61.8)
Q36. People who got the chickenpox vaccine are immunized against mpox (Answer: No).	Yes	133 (31.7)
	No	286 (68.3)
Q37. There is a specific treatment for mpox (Answer: No).	Yes	80 (19.1)
	No	339 (80.9)
Mean score (±SD)	1.38 (±0.19)	

### 3.4 Attitudes of Healthcare Workers Toward Monkeypox Virus Infection

**Figure 3:**
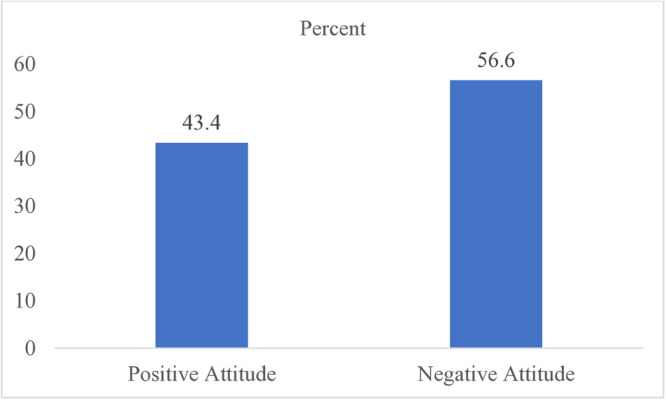
Attitude‐related characteristics of the healthcare workers towards monkeypox virus infection in Central Ethiopia region, 2025 (*n* = 419).

Of the 419 study respondents, 182 (43.4%) (95% CI: 39.1–48) had positive attitudes (Figure 3).

Less than half of the HCWs (180, 42%) strongly agreed that they felt anxious about mpox potentially becoming a global pandemic. Additionally, more than one‐fourth of the HCWs (142, 33.9%) expressed confidence that the Ethiopian Ministry of Health (EMOH) and the local population could effectively control mpox within the country. Less than two‐thirds (263, 62.8%) strongly agreed that mpox could be transmitted to Ethiopia, while 212 (51.8%) strongly agreed that media coverage could positively influence global mpox prevention. Furthermore, 297 (70.9%) HCWs showed interest in learning about travel medicine. Almost two‐thirds (276, 65.9%) strongly agreed that traveling to countries experiencing a monkeypox outbreak poses a risk. One‐fourth (106; 25.3%) strongly agreed that the global spread of mpox could be effectively controlled. A majority of HCWs strongly agreed that they had adequate information about mpox (292, 69.7%) and about the epidemiology of newly emerging diseases (311, 74.2%) (Table 4).

**Table 4:** Attitude‐related characteristics of the healthcare workers toward monkeypox virus infection in Central Ethiopia region, 2025 (*n* = 419).

	Strongly agree	Agree	Neutral	Disagree	Strongly disagree
Attitudes questions	*n* (%)	*n* (%)	*n* (%)	*n* (%)	*n* (%)
Q1. I am confident that the global community can contain the spread of monkeypox.	206 (49.2)	134 (32.0)	60 (14.3)	19 (4.5)	0 (0.0)
Q2. I am confident that the Ethiopian Ministry of Health and the local population can effectively control monkeypox within the country.	142 (33.9)	134 (32.0)	91 (21.7)	39 (9.3)	13 (3.1)
Q3. I feel anxious that monkeypox could become a worldwide pandemic.	180 (42.0)	112 (26.7)	41 (9.8)	53 (12.6)	33 (7.9)
Q4. I believe that monkeypox can place an additional burden on the healthcare systems of affected countries.	256 (61.1)	109 (26)	33 (7.9)	7 (1.7)	14 (3.3)
Q5. I am worried that monkeypox could be transmitted to Ethiopia.	263 (62.8)	122 (29.1)	14 (3.3)	13 (3.1)	7 (1.7)
Q6. Mass media coverage of monkeypox can positively influence its prevention globally.	212 (51.8)	135 (32.2)	34 (8.1)	13 (3.1)	20 (4.8)
Q7. I am interested in learning more about travel medicine.	297 (70.9)	95 (22.7)	7 (1.7)	13 (3.1)	7 (1.7)
Q8. It is risky to travel to countries experiencing a monkeypox outbreak.	276 (65.9)	88 (21)	28 (6.7)	27 (6.4)	0 (0.0)
Q9. The current prevention and control measures for monkeypox are sufficient.	80 (19.1)	61 (14.1)	72 (17.2)	120 (28.6)	86 (20.5)
Q10. I am confident that the spread of monkeypox infection can be effectively controlled globally.	106 (25.3)	167 (39.9)	52 (12.4)	54 (12.9)	40 (9.5)
Q11. I will avoid contact with animals that could harbor the monkeypox virus.	252 (60.1)	86 (20.5)	67 (16.0)	7 (1.7)	7 (1.7)
Q12. I am willing to manage monkeypox‐infected patients as a frontline healthcare provider.	218 (52)	127 (30.3)	61 (14.6)	13 (3.1)	0 (0.0)
Q13. I am confident in my ability to maintain standard precautions to prevent the transmission of monkeypox.	191 (45.6)	148 (35.3)	48 (11.5)	32 (7.6)	0 (0.0)
Q14. Adequate information about the monkeypox virus is essential for healthcare workers.	292 (69.7)	61 (14.6)	33 (7.9)	27 (6.4)	6 (1.4)
Q15. Travel to countries affected by monkeypox should be restricted to prevent its spread.	169 (40.3)	102 (24.3)	107 (25.5)	21 (5.0)	20 (4.8)
Q16. If I become infected with monkeypox, I will follow medical advice and adhere to isolation guidelines.	277 (66.1)	75 (17.9)	46 (11.0)	21 (5.0)	0 (0.0)
Q17. Proper health education and safe patient handling are crucial to preventing transmission of the monkeypox virus between patients and healthcare workers.	298 (71.1)	54 (12.9)	61 (14.6)	6 (1.4)	0 (0.0)
Q18. Proper counseling by health workers to patients and attendants can help reduce the prevalence of viral diseases like monkeypox.	291 (69.5)	81 (19.3)	20 (4.8)	27 (6.4)	0 (0.0)
Q19. I want to receive training on monkeypox before any new cases are detected in our country.	250 (59.7)	81 (19.3)	60 (14.3)	21 (5.0)	7 (1.7)
Q20. I am willing to take the monkeypox vaccine if it becomes available.	275 (65.6)	69 (16.5)	48 (11.5)	20 (4.8)	7 (1.7)
Q21. Healthcare workers should be tested when they are in contact with someone infected.	242 (57.8)	102 (24.3)	27 (6.4)	35 (8.4)	13 (3.1)
Q22. I am willing to visit family members or friends who are infected with monkeypox.	120 (28.6)	120 (28.6)	93 (22.2)	41 (9.8)	45 (10.7)
Q23. I should practice more hygienic preventive measures because of the risk of monkeypox.	257 (61.3)	114 (27.2)	27 (6.4)	21 (5.0)	0 (0.0)
Q24. All individuals with a skin rash should be tested for monkeypox.	157 (37.5)	141 (33.7)	61 (14.6)	27 (6.4)	33 (7.9)
Q25. I am concerned that monkeypox could become a new pandemic with an impact similar to COVID‐19.	157 (37.5)	122 (29.1)	61 (14.6)	52 (12.4)	27 (6.4)
Q26. I am interested in learning more about monkeypox.	330 (78.8)	55 (13.1)	20 (4.8)	14 (3.3)	0 (0.0)
Q27. I am interested in learning more about the epidemiology of newly emerging diseases.	311 (74.2)	68 (16.2)	27 (6.4)	13 (3.1)	0 (0.0)
Mean score (±SD)	1.79 (±0.51)				

### 3.5 Factors Associated With the Knowledge and Attitudes of Healthcare Workers Toward Monkeypox Virus Infection

#### 3.5.1 Factors Associated With the Knowledge of Healthcare Workers Toward Monkeypox Virus Infection

Variables such as residence, education level, work experience, profession type, health facility level, monthly income (ETB/USD), whether you had heard of mpox before, the first time you heard of mpox, and attitude were entered into the bivariable logistic regression model. After adjustment in the multivariable logistic regression analysis, education level, work experience, profession type, monthly income, and attitude were significantly associated with good knowledge (*p* value < 0.005).

Healthcare workers with a master's degree or higher were 3.46 times more likely to have a good knowledge than those with a diploma (AOR = 3.46; 95% CI: 1.60–7.47). HCWs who were health officers and pharmacists were 7.00 and 2.68 times more significantly associated with a good knowledge than nurses/midwives (AOR = 7.00; 95% CI: 3.20–15.32 and AOR = 2.68; 95% CI: 1.17–6.13, respectively). Those with low and medium monthly incomes were 5.15 and 2.35 times more significantly associated with a good knowledge than those with high monthly incomes (AOR = 5.15; 95% CI: 2.02–13.10 and AOR = 2.35; 95% CI: 1.32–4.20, respectively). Furthermore, HCWs with a positive attitude toward mpox were 2.11 times more likely to have good knowledge than those with a negative attitude (AOR = 2.11; 95% CI: 1.25–3.59). In contrast, HCWs with 5–10 years of work experience were 87% less likely to be significantly associated with a good knowledge than with ≥ 10 years of experience (AOR = 0.13; 95% CI: 0.07–0.24) (Table 5).

**Table 5:** Factors associated with healthcare worker knowledge of monkeypox virus infection in Central Ethiopia region, 2025 (*n* = 419).

	Knowledge of HCWs toward the mpox Virus Infection				
Variable	Poor	Good	COR (95% CI)	*p*	AOR (95% CI)
Residence					
Urban	176 (78.9)	131 (73.3)	1		
Rural	47 (21.1)	65 (33.2)	1.86 (1.20–2.88)	0.006	
Educational level					
Diploma	87 (39.0)	59 (30.1)	1	1	1
Bachelor degree	65 (29.1)	71 (36.2)	1.61 (1.01–2.58)	0.048	0.96 (0.53–1.75)
Master's degree and above	71 (31.8)	66 (33.7)	1.37 (0.86–2.20)	0.189	3.46 (1.60–7.47)*
Work experience (in years)					
≤ 5	34 (15.2)	73 (37.2)	1.72 (1.04–2.84)	0.035	0.99 (0.53–1.86)
5–10	109 (48.9)	23 (11.7)	0.17 (0.10–0.29)	0.001	0.13 (0.07–0.24)*
≥ 10	80 (35.9)	100 (51.0)	1		1
Profession type					
Laboratory technician/technologist	31 (13.9)	11 (5.6)	0.48 (0.23–1.03)	0.060	0.59 (0.45–1.42)
Nurse/midwife	87 (39.0)	64 (32.7)	1		1
Health officer	18 (8.1)	54 (27.6)	4.08 (2.19–7.61)	0.001	7 (3.20–15.32)*
Pharmacist	20 (9.0)	20 (10.2)	1.36 (0.68–2.73)	0.389	2.68 (1.17–6.13)*
Environmental health	18 (8.1)	6 (3.1)	0.45 (0.17–1.21)	0.113	0.88 (0.29–2.66)
Medical doctor (general practitioner or specialist)	36 (16.1)	31 (15.8)	1.17 (0.66–2.09)	0.594	0.98 (0.41–2.34)
Others	13 (5.8)	10 (5.1)	1.05 (0.43–2.54)	0.921	0.71 (0.24–2.09)
Level of the health facility					
Health post/health Center	55 (24.7)	75 (38.3)	1.69 (1.05–2.73)	0.030	
Primary hospital/general hospital	86 (38.6)	55 (28.1)	0.80 (0.50–1.27)	0.336	
Referral/specialized hospital	82 (36.8)	66 (33.7)	1		
Monthly income					
< 7424 ETB (≈ < 53.88 USD) (low)	20 (9.0)	28 (14.3)	1.85 (0.98–3.51)	0.060	5.15 (2.02–13.10)*
7424–10,129 ETB (≈ 53.88–73.51 USD) (medium)	92 (41.3)	84 (42.9)	1.21 (0.80–1.82)	0.369	2.35 (1.32–4.20)*
≥ 10,130 ETB (≈ ≥ 73.51 USD) (high)	111 (49.8)	84 (42.9)	1		1
Heard about the mpox virus before?					
Yes	152 (68.2)	144 (73.5)	1		
No	71 (31.8)	52 (26.5)	0.77 (0.51–1.18)	0.234	
First time you heard about mpox					
I did not hear about it	14 (6.3)	20 (10.2)	1.56 (0.76–3.20)	0.230	
Within several days or weeks ago	62 (27.8)	41 (20.9)	0.72 (0.46–1.14)	0.160	
Within the last month or so	147 (65.9)	135 (68.9)	1		
Attitude					
Negative	119 (53.4)	118 (60.2)	1		1
Positive	104 (46.6)	78 (39.8)	1.32 (0.90–1.95)	0.159	2.11 (1.25–3.59)*

*Note:* Where: Others: 13 psychiatrists, 10 health informaticists, COR is the crude odds ratio, AOR is the adjusted odds ratio, 1 is the reference.

* The variable significance is at a *p* value < 0.05 in the multivariable analysis.

#### 3.5.2 Factors Associated With the Attitudes of Healthcare Workers Toward Monkeypox Virus Infection

Variables such as gender, age, education level, work experience, profession type, information about mpox virus infection during medical education, current information about mpox virus infection, and knowledge were entered into the bivariable logistic regression model. After adjustment in the multivariable logistic regression analysis, gender, age, profession type, information about mpox during medical education, and current information about mpox infection were significantly associated with a positive attitude toward mpox at a *p* value < 0.005.

Healthcare workers who were medical doctors were 4.78 times more likely to be significantly positively associated with attitude than nurses/midwives (AOR = 4.78; 95% CI: 2.39–9.55). Those who had received information about mpox infection during their medical education were 3.29 times more likely to have a significantly positive attitude than those who did not (AOR = 3.29; 95% CI: 1.91–5.67). Conversely, female HCWs were 82% less likely to be significantly positively associated with a positive attitude than their male counterparts (AOR = 0.18; 95% CI: 0.08–0.41). Similarly, HCWs aged ≥ 32 years were 55% less likely to be significantly positively associated with a positive attitude than those aged < 32 (AOR = 0.45; 95% CI: 0.28–0.73). In addition, HCWs who lacked current information about mpox infection were 53% less likely to be significantly positively associated with a positive attitude than those with such information (AOR = 0.47; 95% CI: 0.26–0.88) (Table 6).

**Table 6:** Factors associated with healthcare worker attitudes toward monkeypox virus infection in Central Ethiopia region, 2025 (*n* = 419).

	Attitude of HCWs toward the mpox Virus infection				
Variable	Poor	Good	COR (95% CI)	*p*	AOR (95% CI)
Gender					
Male	190 (80.2)	169 (92.9)	1	1	1
Female	47 (19.8)	13 (7.1)	0.31 (0.16–0.60)	0.001	0.18 (0.08–0.41)*
Age in years					
< 32	118 (49.8)	114 (62.6)	1	1	1
≥ 32	119 (50.2)	68 (37.4)	0.59 (0.40–0.88)	0.009	0.45 (0.28–0.73)*
Educational level					
Diploma	90 (38.0)	56 (30.8)	1		
Bachelor degree	94 (39.7)	42 (23.1)	0.72 (0.44–1.18)	0.188	
Master's degree and above	53 (22.4)	84 (46.2)	2.55 (1.58–4.11)	0.001	
Work experience (in years)					
≤ 5	53 (22.4)	54 (29.7)	1.68 (1.03–2.72)	0.036	
5–10	72 (30.4)	60 (33.0)	1.37 (0.87–2.17)	0.174	
≥ 10	112 (47.3)	68 (37.4)	1		
Profession type					
Laboratory technician/technologist	25 (10.5)	17 (9.3)	1.30 (0.64–2.61)	0.471	1.75 (0.76–4.04)
Nurse/midwife	99 (41.8)	52 (28.6)	1		1
Health officer	51 (21.5)	21 (11.5)	0.78 (0.43–1.44)	0.433	0.61 (0.31–1.21)
Pharmacist	18 (7.6)	22 (12.1)	2.33 (1.15–4.72)	0.019	1.66 (0.75–3.64)
Environmental health	16 (6.8)	8 (4.4)	0.95 (0.38–2.37)	0.916	0.64 (0.23–1.81)
Medical doctor (general practitioner or specialist)	17 (7.2)	50 (27.5)	5.60 (2.94–10.70)	0.001	4.78 (2.39–9.55)*
Others	11 (4.6)	12 (6.6)	2.08 (0.86–5.03)	0.105	1.50 (0.55–4.10)
Information about the mpox virus infection during your medical education					
Yes	47 (19.8)	92 (50.5)	4.13 (2.68–6.36)	0.001	3.29 (1.91, 5.67)*
No	190 (80.2)	90 (49.5)	1	1	1
Current information about the mpox virus infection					
Yes	132 (55.7)	154 (84.6)	1	1	1
No	105 (44.3)	28 (15.4)	0.23 (0.14–0.37)	0.001	0.47 (0.26–0.88)*
Knowledge					
Poor	119 (50.2)	104 (57.1)	1		
Good	118 (49.8)	78 (42.9)	1.32 (0.90–1.95)	0.159	

*Note:* Where: COR is the crude odds ratio, AOR is the adjusted odds ratio, and 1 is the reference.

* The variable significance at a *p* value < 0.05 in the multivariable analysis.

## 4 Discussion

Healthcare workers are at the frontline of preventing and managing emerging and re‐emerging infectious diseases such as mpox, and their knowledge and attitudes directly affect early detection, reporting, and infection control efforts [1, 38, 39]. There have been reports of an mpox outbreak in Ethiopia, but none in the central Ethiopia region. However, the area is vulnerable because of its shared border region, where cases have been documented [19]. This highlights the potential risk of local mpox transmission from neighboring areas. The present study assessed HCWs' knowledge and attitudes towards mpox.

Of the 419 study respondents, only 46.8% (95% CI: 42.0–51.7) of HCWs had good knowledge of mpox, indicating a substantial knowledge gap among HCWs regarding this infectious disease. One potential reason for this limited knowledge may be the lack of exposure to mpox‐related training and education. In this study, most participants (373, 89%) had not received any training on mpox infection. Additionally, 413 (98.6%) had not attended national conferences, and none of the 419 (100%) had participated in international mpox‐related conferences. Furthermore, 280 (66.8%) reported not receiving information about mpox during their medical education. Most HCWs (409, 97.6%) stated no confirmed mpox infection cases in the Central Ethiopia Region. This study's findings reveal that many HCWs lack awareness of the similarities in signs and symptoms between mpox, chickenpox, and smallpox, with (226, 53.9%) and (205, 48.9%) not recognizing these connections, respectively. Additionally, 222 (53%) were unaware that antiviral medications are necessary for managing mpox. Misconceptions about mpox persist, with (221, 52.7%) of HCWs incorrectly believing that a specific vaccine exists, and (339, 80.9%) thinking there is a distinct treatment available. These statistics indicate significant gaps in knowledge critical for the timely detection and management of mpox. The diminished focus on diseases like smallpox and chickenpox in medical education may play a role in this lack of awareness, underscoring the need for enhanced training in similar conditions. This underscores the urgent need to revise educational resources and implement targeted health education programs to improve HCWs' knowledge of emerging and re‐emerging infectious diseases, such as mpox.

This study finding is comparable to those study from the University of Gondar Comprehensive Specialized Referral Hospital, Ethiopia (48.40%) [28], Southwestern Saudi Arabia (44.1%) [32], Ohio, United States (48.9%) [31], and a systematic review and meta‐analysis (SRMA) conducted on the world's population towards monkeypox and its vaccines (46.6%) [40]. However, it is higher than findings from studies conducted in Addis Ababa, Ethiopia (35.4%) [41], Northwest Ethiopia (38.5%) [1], global SRMA study conducted among HCWs (26%) [42], SRMA study conducted among knowledge‐attitudes (KAs) towards mpox (33%) [43], the Kurdistan Region of Iraq (18.2%) [11], Indonesia (36.5%) [12], and the general population in Pakistan (34.4%) [13]. Moreover, this finding is lower than those reported in studies among Bangladeshi nurses (57.97%) [44], in Nigeria (58.7%) [2], among physicians in Saudi Arabia (55%) [5], in Egypt (55.3%) [8], another study in Nigeria (60.5%) [45], the general population in Nepal (53.8%) [46], and in Cameroon (42.1%) [33]. Differences in the study setting, study period, attitudes toward mpox infection, sources of information, the accessibility and availability of training programs, sociocultural differences, and educational curricula between countries. Could be all contributing factors to this discrepancy. The lack of endemic mpox infection in the Central Ethiopia Region likely contributes to the limited awareness among HCWs.

Notably, HCWs with a master's degree or higher exhibited significantly greater knowledge compared to those with only a diploma, reflecting findings from studies in Ethiopia [1, 28], Bangladesh [44], Nigeria [2], and Pakistan [13] that link higher education levels to improved knowledge in this domain. This result may be due to higher education enhancing health awareness, critical thinking, and the ability to interpret health information. Healthcare workers with advanced qualifications are more likely to access credible sources, engage in training and research, and develop effective information‐seeking habits. These factors contribute to a more comprehensive understanding of mpox and improved knowledge levels.

Healthcare workers, including health officers and pharmacists, had significantly greater knowledge than nurses/midwives. This finding is supported by studies conducted at the University of Gondar Comprehensive Specialized Referral Hospital in Ethiopia [28] and in Nigeria [45], which reported a significant association between occupation and knowledge level among healthcare workers. This could be attributed to health officers and pharmacists receiving more specialized training and education on disease management and therapeutic interventions. In the Ethiopian context, health officers are primarily responsible for diagnosis and comprehensive disease management, including emerging infections such as mpox, particularly when physicians are unavailable. Similarly, pharmacists are involved in drug selection, preparation, and management and are often required to stay up to date on treatment guidelines, including those related to mpox. Also, a pharmacist is the ultimate expert on the medicinal product/medicines, as he has a robust academic background in chemistry, biology, pharmacology, pharmacotherapy, pharmacokinetics, toxicology, pharmaceutical technology, regulatory affairs, physiology, and pathophysiology, among others. In contrast, nurses and midwives mainly focus on delivering direct patient care and routine clinical support. As a result, they may have fewer opportunities to engage with the diagnostic and pharmacological aspects of complex diseases like mpox, which may contribute to their relatively lower knowledge levels [28].

Healthcare workers with low and medium monthly incomes were significantly more likely to have a good knowledge than those with high monthly incomes. This finding contrasts with studies conducted in Bangladesh [44]. One possible explanation is that low‐ and medium‐income HCWs are more likely to be engaged in frontline clinical roles, where they have greater exposure to patients, infectious disease cases, and routine in‐service training [47, 48]. In contrast, higher‐income HCWs may more often occupy administrative or supervisory positions with reduced direct clinical involvement and fewer opportunities for regular training [49]. Thus, differences in clinical engagement and access to ongoing professional development, rather than income itself, may explain the observed variation.

Furthermore, HCWs with a positive attitude toward mpox were significantly more likely to have a good knowledge than those with a negative attitude. This finding was supported by a study among Bangladeshi nurses, which found a strong positive correlation between strong knowledge and a positive attitude toward mpox [44]. This might be due to HCWs with good feelings or beliefs having good awareness and understanding; they want to learn more about mpox. This thinking makes them more willing to look at health education materials or learn about mpox prevention and control methods.

Healthcare workers with 5–10 years of work experience exhibit a notably higher level of knowledge than those with 10 or more years. This finding was consistent across studies from Ethiopia [28, 41], a global SRMA [42], and Southern Italy [15]. One possible explanation is that those with longer experience may have more exposure to outbreaks, institutional protocols, and training, which enhances their awareness of emerging infections [28, 50]. In contrast, mid‐career HCWs often face heavier clinical workloads and may have fewer opportunities for continuous professional development, potentially limiting their access to updated information. Thus, differences in training exposure and evolving professional roles across career stages, rather than experience alone, may explain the observed variation.

This study revealed that only 43.4% (95% CI: 39.1–48.0) of HCWs had a positive attitude toward mpox, indicating that a significant proportion had a less favorable outlook on the disease. One potential reason for this limited positive attitude may be that only 167 (39.9%) of HCWs agreed that the global spread of monkeypox can be effectively controlled, despite the disease being declared a public health emergency of international concern [2, 6, 8, 9, 10]. Additionally, 223 (53.2%) of HCWs demonstrated poor knowledge about mpox, which may have contributed to their negative attitudes. However, fostering positive attitudes among HCWs is critical for effective disease control and prevention, as it encourages active participation in surveillance, reporting, and the implementation of preventive measures.

The finding of this study was lower than those reported in Northwest Ethiopia (62%) [1], from University of Gondar Comprehensive Specialized Referral Hospital, Ethiopia (49.2%) [28], among Bangladeshi nurses (93.12%) [44], in Southwestern Saudi Arabia (percentage not specified) [32], in the Kurdistan Region of Iraq (50.1%) [11], and in a SRMA conducted on the global population regarding monkeypox and its vaccines (71.9%) [40]. The findings reveal notable differences compared to a global SRMA of healthcare workers, which reported a prevalence of (34.6%) [42] and a study of key populations regarding mpox showing (40%) [43]. The results also align with research from Egypt at (44.5%) [8] and Pakistan at (41.7%) [13] among the general population. These variations may be due to factors such as differing definitions of attitudes, socioeconomic and cultural contexts, access to information, sample sizes, study designs, and the populations studied.

Female HCWs were significantly less likely to be positively associated with a positive attitude than their male counterparts. This finding is similar to a study conducted in Egypt [8], but contrasts with a study conducted in Bangladesh [44], suggesting context‐specific variation. One possible explanation could be related to differences in perceived occupational risk, access to information, or sociocultural factors influencing risk perception and health attitudes within the study setting. Variations in professional roles or workplace exposure contribute to this difference.

Healthcare workers aged ≥ 32 years were significantly less likely to have a positive attitude than those aged < 32 years. This finding was similar to that of a study conducted among Bangladeshi nurses [44], in contrast with a study conducted in Northwest Ethiopia [1]. The younger generation is more familiar with the Internet and therefore has better access to information regarding mpox, which is mainly available online. In addition, older doctors may rely more on experience than information from other sources [12, 51].

Medical doctors were significantly more likely than nurses/midwives to have a positive attitude. Their specialized training, education curriculum, and job description might be the reason. A physician mainly focuses on diagnostics and comprehensive disease management, including mpox. Additionally, during these kinds of endemics, Physicians may have the opportunity to attend conferences that provide up‐to‐date information on the status of global disease knowledge. On the other hand, nurses prioritize providing direct patient care, which would restrict their exposure to mpox [28]. This is likely because it is expected to optimize their ability to manage potential cases.

Healthcare workers who had information about mpox during their medical education were significantly more likely to have a positive attitude than their counterparts. This finding was similar to the study conducted in Nepal [46]. It demonstrated that those who studied monkeypox during their academic life had a significantly more positive attitude toward controlling and preventing mpox (*p* = 0.025). This observation highlights that HCWs with access to information about mpox exhibit a higher level of awareness than those who lack such information. Current knowledge regarding its transmission, symptoms, prevention, and treatment is crucial for HCWs. Research shows that those without up‐to‐date information are significantly less likely to maintain a positive attitude toward managing mpox, a finding echoed by a study in Northwest Ethiopia [1]. This underscores that outdated or insufficient information can lead to misconceptions and fear, ultimately hindering effective prevention and control efforts.

### 4.1 Key Knowledge Items and Recommended Actions for Healthcare Workers on Monkeypox

#### 4.1.1 Key Knowledge Items

- Monkeypox is a zoonotic viral disease caused by the monkeypox virus, a member of the Orthopoxvirus genus.
- Human‐to‐human transmission occurs through contact with lesions, body fluids, respiratory droplets, and contaminated materials.
- Key symptoms include fever, headache, muscle aches, lymphadenopathy, and a characteristic rash that progresses from macules to pustules.
- The incubation period is typically 6–13 days (range: 5–21 days).
- Early recognition and isolation of suspected cases are critical to prevent nosocomial spread.
- Supportive care and symptom management are the mainstay treatments, as no specific antiviral is widely available.
- The smallpox vaccine provides cross‐protection and is recommended for at‐risk healthcare workers.

#### 4.1.2 Recommended Actions for the Health System

**Figure 4:**
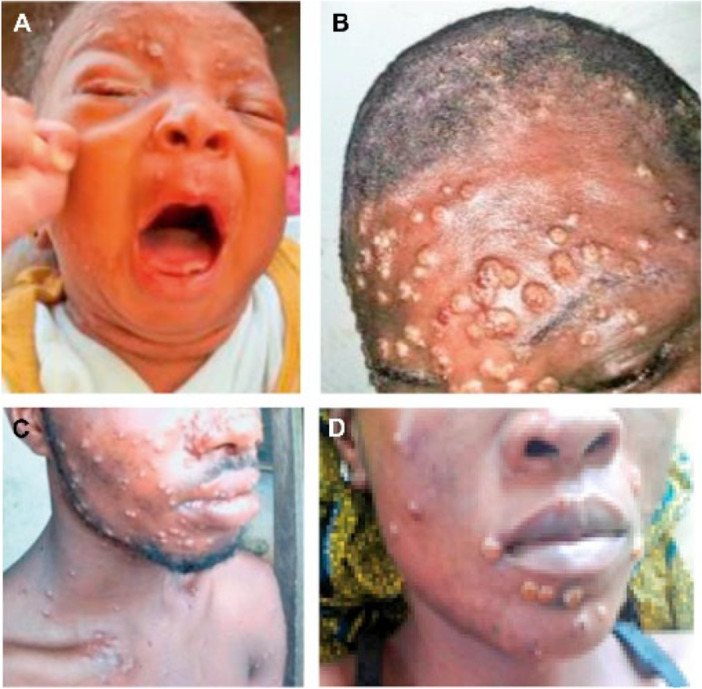
Monkeypox lesions on face (for educational purposes). (A) Neonate with multiple vesiculopustular lesions involving the face and scalp, consistent with congenital or perinatal mpox infection. (B) Close‐up view of the scalp and forehead showing numerous umbilicated vesiculopustular lesions at different stages of development. (C) Adult male with multiple papulovesicular and pustular lesions distributed over the face, neck, and upper chest, characteristic of mpox. (D) Adult female with localized umbilicated papules and pustules involving the perioral region and chin.

▪ Conduct training programs on monkeypox case identification and infection prevention measures for healthcare workers.
▪ Implement public health campaigns to raise community awareness of monkeypox symptoms and prevention measures.
▪ Ensure the availability of personal protective equipment (PPE) in all public health facilities.
▪ Establish protocols for early isolation, case management, and contact tracing of suspected cases.
▪ Promote targeted vaccination campaigns for healthcare workers and high‐risk groups.
▪ Strengthen surveillance systems to detect and report monkeypox cases (Figure 4) promptly.

## 5 Strengths and Limitations

This study is the first of its kind in the Central Ethiopia Region to assess healthcare workers' knowledge and attitudes toward the re‐emergent human mpox virus. It also compared knowledge and attitudes across different healthcare professional categories. The study revealed significant gaps in both knowledge and attitudes among the participants. These findings have important implications for program managers implementing effective interventions and offer valuable insights for future researchers.

However, several limitations should be considered when interpreting the results. First, due to the cross‐sectional study design, causal inferences cannot be established. Second, convenience sampling introduced selection bias, potentially overrepresenting younger, urban, and digitally connected healthcare workers while underrepresenting those with limited internet access. Additionally, healthcare professionals from private health institutions were excluded, potentially limiting the sample's representativeness. Furthermore, the gender distribution of participants was highly skewed toward males, which may have influenced the findings on attitudes. These factors collectively limit the generalizability of the results. Thus, the findings should be interpreted with caution and not assumed to represent all healthcare workers in the Central Ethiopia Region.

Second, relying on self‐reported data through an online survey may have introduced recall and social desirability biases, potentially affecting the accuracy of the reported knowledge and attitudes. Moreover, the cross‐sectional study's design provides only a snapshot of the participants' knowledge and attitudes simultaneously. Lastly, surveying English posed a language barrier for some participants, potentially affecting their comprehension and responses.

## 6 Conclusions and Recommendations

The good knowledge and positive attitudes toward mpox among HCWs in this study were relatively low. Variables such as gender, age, educational level, monthly income (ETB/USD), work experience, profession type, exposure to information about mpox during medical education, access to current information on mpox, and having a positive attitude were significantly associated with HCWs' knowledge and attitudes. The findings highlight the limited knowledge and attitudes among HCWs regarding mpox and underscore the regional need for targeted improvement. Health facilities and universities, both at the regional and national levels, should take active measures to enhance HCWs' knowledge and attitudes by providing opportunities for continuing education and organizing training sessions, seminars, conferences, webinars, and awareness campaigns. Additionally, medical curricula should be updated to address re‐emerging infectious diseases, such as mpox, comprehensively. Moreover, future qualitative research is recommended to provide a deeper, more holistic understanding of the factors influencing HCWs' knowledge and attitudes toward mpox in the Central Ethiopia Region.

## Author Contributions

**Yilma Markos Larebo:** conceptualization, data curation, formal analysis, funding acquisition, investigation, methodology, project administration, resources, software, supervision, visualization, validation, writing – original draft, writing – review and editing. **Zerfework Debebe Argago:** conceptualization, data curation, funding acquisition, investigation, methodology, resources, validation, visualization, writing – original draft, writing – review and editing. **Markos Selamu Jifar:** conceptualization, investigation, funding acquisition, writing – original draft, writing – review and editing, methodology, validation, visualization, data curation, resources. **Abebe Alemu Anshebo:** data curation, resources, methodology, validation, visualization, writing – review and editing, conceptualization, investigation, funding acquisition, writing – original draft. **Sujit Kumar Behera:** conceptualization, data curation, funding acquisition, investigation, methodology, resources, supervision, writing – original draft, writing – review and editing, validation, visualization. **Natarajan Gopalan:** data curation, supervision, resources, methodology, validation, visualization, writing – review and editing, writing – original draft, funding acquisition, investigation, conceptualization.

## Funding

The authors have nothing to report.

## Consent

The authors have nothing to report.

## Conflicts of Interest

The authors declare no conflicts of interest.

## Transparency Statement

The lead author, Yilma Markos Larebo, affirms that this manuscript is an honest, accurate, and transparent account of the study being reported; that no important aspects of the study have been omitted; and that any discrepancies from the study as planned (and, if relevant, registered) have been explained.

## Supporting information


Supporting File

## Data Availability

The data are available in the Supporting Information for this article.
