# Supplementary material for: Knowledge and Attitudes of Healthcare Workers Toward the Re‐Emergence of Human Monkeypox Virus Infection in Public Health Facilities in the Central Ethiopia Region: An Online Cross‐Sectional Study
Source: Health Sci Rep. 2026 Jul 30;9(8):e72854. doi: 10.1002/hsr2.72854 (PMC13421795; doi:10.1002/hsr2.72854)
Supplement: Supplementary file 1 — Supporting File [file HSR2-9-e72854-s001.pdf]

Participant Information Sheet and Consent Form

Title of the Study: Knowledge and Attitudes of Healthcare Workers Toward the Re-Emergence of Human Monkeypox Virus Infection in Public Health Facilities in the Central Ethiopia Region: An Online Cross-Sectional Study

Introduction

You are invited to participate in a research study about the knowledge and attitudes of healthcare workers toward the re-emergence of the human monkeypox virus infection. Your participation is entirely voluntary. Before you decide, please read the following information carefully.

Purpose of the Study

This study aims to assess healthcare workers' knowledge and attitudes regarding the re-emergence of the human monkeypox virus infection to inform prevention and control measures and enhance community awareness and service delivery. The survey will take approximately 15 to 20 minutes to complete. Your responses will be recorded anonymously. Your name or any other identifying information will not be collected. You may skip any question or stop the survey at any time without penalty.

Risks and Benefits

There are no foreseeable risks to your participation in this survey. Your input will provide valuable insights for improving public health preparedness and response to monkeypox virus infection.

Confidentiality

All information collected in this study will be kept strictly confidential. Data will be stored securely and used only for research purposes. Only the research team will have access to the anonymized data.

Voluntary Participation and Right to Withdraw

Your participation is voluntary. You may refuse to participate or withdraw at any time without any

negative consequences and without giving a reason.

Whom to Contact

You may contact us with any questions or concerns about this study. Yilma Markos Lerbo: Phone: +215917189182 and Email: yilmamark@gmail.com.

Consent Statement

Do you agree to participate in this study voluntarily?

☐ Yes

☐ No (If no, terminate the survey and say thank you)

By selecting "Yes" (or signing below, if applicable), you confirm that you have read and understood the information provided above, had the opportunity to ask questions, and agree to participate in the study.

Name of Participant (Optional):

____

Participant Signature:

______________________ **Date:** ___ / ___ / _____

* Indicates required question

1. Do you have permission to continue? *

Mark only one oval.

- ◯ Yes *Skip to question 2*
- ◯ No

Sociodemographic-related characteristics of the health workers towards preventing and controlling the re-emergence of human monkeypox infection in the central Ethiopia region.

2. What is your gender? *

Check all that apply.

- [ ] Male
- [ ] Female
- [ ] Other: ______________________

3. What is your age (in complete years)? *

____

4. What is your employer location or place of residence? *

Mark only one oval.

- ( ) Urban
- ( ) Rural

5. Which zone do you reside or work in within the Central Ethiopia Region? *
Please write the name of your zone.

____

6. What is your marital status? *

Mark only one oval.

- ( ) Single
- ( ) Married
- ( ) Divorced
- ( ) Widowed
- ( ) Separated
- ( ) Other: ______________________

7. What is the highest level of education you have completed? *

Mark only one oval.

- ( ) Diploma
- ( ) Bachelor's degree
- ( ) Master's degree
- ( ) Doctorate (PhD or equivalent)
- ( ) Other: ______________________

8. What is your religion? *

Mark only one oval.

- ( ) Orthodox
- ( ) Muslim
- ( ) Protestant
- ( ) Catholic
- ( ) I have no religion
- ( ) Other: ______________________

9. How many years of work experience do you have? *

Please enter your total work experience in complete years only:

____

10. What is your profession? *

Mark only one oval.

- Laboratory Technician/Technologist
- Nurse
- Midwife
- Health Officer
- Pharmacist/Druggist
- Environmental Health Professional
- Medical Doctor (General Practitioner or Specialist)
- Other: ____________________

11. What is the level of the health facility where you are currently working? *

Mark only one oval.

- Health Post
- Health Center
- Primary Hospital
- General Hospital
- Referral/Specialized Hospital
- Other: ____________________

12. What is your total monthly salary (in Ethiopian Birr)? *

____

13. Have you received any training on monkeypox (mpox) before?

Mark only one oval.

- Yes
- No

14. If yes, please specify the type or source of the training:

____

15. Have you ever attended any national conferences related to mpox virus infection?

Mark only one oval.

- Yes
- No

16. If yes, please specify the name of the conference (if known):

____

17. Have you ever attended any international conferences related to mpox virus infection?

Mark only one oval.

- Yes
- No

18. If yes, please specify the name of the conference (if known):

____

19. Have you heard about the monkeypox virus before? *

Mark only one oval.

- Yes
- No *Skip to question 22*

20. If yes to the previous question, what were your sources of information about mpox virus infection during your medical education?

You may select more than one option.

Mark only one oval per row.

	Column 1
**Television/ Radio**	⬭
**Newspapers/magazines**	⬭
**Health workers/ Health care providers**	⬭
**Social media**	⬭
**Internet**	⬭
**E-mail message**	⬭
**Grocery store**	⬭
**Their friends/ Family /friends/neighbour**	⬭
**Religious leaders/teachers**	⬭
**Mpx info. leaflets/ brochures/ posters**	⬭
**Others**	⬭

21. When did you first hear about mpox? *

Please specify the approximate year or time period:

____

Knowledge-Related Characteristics of Healthcare Workers Towards the Prevention and Control of the Re-emergence of Human Monkeypox Infection in the Central Ethiopia Region.

22. Are there any confirmed human monkeypox cases in Ethiopia? *

Mark only one oval.

- Yes
- No
- I don’t know

23. Are there any confirmed human mpox cases in the Central Ethiopia Region? *

Mark only one oval.

- Yes
- No
- I don’t know

24. Is monkeypox (mpox) currently prevalent in Middle Eastern countries? *

The term "**Middle Eastern countries**" generally refers to a region that spans parts of **Western Asia and North Africa.** Includes Bahrain, Cyprus, Egypt, Iran, Iraq, Israel, Jordan, Kuwait, Lebanon, Oman, Palestine (West Bank and Gaza Strip), Qatar, Saudi Arabia, Syria, Turkey, the United Arab Emirates (UAE), and Yemen. Sometimes Afghanistan, Libya, Sudan, and Pakistan.

Mark only one oval.

- Yes
- No
- I don’t know

25. Is monkeypox (mpox) prevalent in Southeast Asian countries? *

The region consists of **11 countries,** like Brunei Darussalam, Cambodia, East Timor (Timor-Leste), Indonesia, Laos (Lao People's Democratic Republic), Malaysia, Myanmar (Burma), the Philippines, Singapore, Thailand, and Vietnam.

Mark only one oval.

- Yes
- No
- I don’t know

26. Is monkeypox (mpox) prevalent in Western and Central Africa? *

There are **16 countries in Western Africa**, including Benin, Burkina Faso, Cape Verde (Cabo Verde), Verde),Côte d'Ivoire (Ivory Coast), The Gambia, Ghana, Guinea, Guinea-Bissau, Liberia, Mali, Mauritania, Niger, Nigeria, Senegal, Sierra Leone, and Togo. There are **9 countries in Central Africa**, including Angola, Cameroon, the Central African Republic, Chad, the Republic of the Congo, the Democratic Republic of the Congo (DRC), Equatorial Guinea, Gabon, and São Tomé and Príncipe.

Mark only one oval.

- Yes
- No
- I don’t know

27. Have there been any confirmed human mpox cases in the USA, Canada, the UK, or Europe? *

Mark only one oval.

- Yes
- No
- I don’t know

28. What is the typical duration of illness for monkeypox (mpox) after symptoms appear? *

Mark only one oval.

- 2–4 days
- 2–4 weeks
- 5–21 days
- I don't know

29. What is the typical incubation period of mpox virus infection? *

Mark only one oval.

- 1–3 days
- 5–21 days
- 2–4 weeks
- I don't know

30. Since 2022, have monkeypox (mpox) cases been reported in more than 130 countries worldwide? *

Mark only one oval.

- Yes
- No
- I don't know

31. Is monkeypox (mpox) caused by a newly discovered virus? *

Mark only one oval.

- Yes
- No
- I don't know

32. Is monkeypox (mpox) considered a re-emerging disease? *

Mark only one oval.

- Yes
- No
- I don’t know

33. Is human monkeypox (mpox) a viral disease? *

Mark only one oval.

- Yes
- No
- I don’t know

34. Is monkeypox (mpox) a bacterial infection? *

Mark only one oval.

- Yes
- No
- I don’t know

35. Can the monkeypox (mpox) virus be spread through contact with an infected person? *

Mark only one oval.

- Yes
- No
- I don’t know

36. Can monkeypox (mpox) be transmitted to humans through direct contact with infected animals? *

Mark only one oval.

- Yes
- No
- I don’t know

37. Can monkeypox (mpox) be transmitted through skin-to-skin contact? *

Mark only one oval.

- Yes
- No

38. Is international travel the main source of imported monkeypox (mpox) cases? *

Mark only one oval.

- Yes
- No
- I don’t know

39. Can monkeypox (mpox) be transmitted through direct contact with body fluids, sores, or contaminated materials (such as clothing, bedding, or surfaces) from an infected person?

Mark only one oval.

- Yes
- No
- I don’t know

40. Can the monkeypox (mpox) virus cross the placenta from an infected mother to her fetus? *

Mark only one oval.

- Yes
- No
- I don’t know

41. Can monkeypox (mpox) spread through intimate contact such as sex, kissing, cuddling, or contact with sores, as well as through droplets from coughing or sneezing? *

Mark only one oval.

- Yes
- No
- I don’t know

42. Is blood-borne transmission of monkeypox (mpox) possible? *

Mark only one oval.

- Yes
- No
- I don’t know

43. Can monkeypox (mpox) be transmitted through eating food contaminated with the virus?

Mark only one oval.

- Yes
- No
- I don’t know

44. Can monkeypox (mpox) be transmitted through the bite of an infected monkey or other animal? *

Mark only one oval.

- Yes
- No
- I don’t know

45. Can monkeypox (mpox) be transmitted by eating insufficiently cooked meat from an infected animal? *

Mark only one oval.

- Yes
- No
- I don’t know

46. Do monkeypox (mpox) and chickenpox have similar signs and symptoms? *

Mark only one oval.

- Yes
- No
- I don’t know

47. Do monkeypox (mpox) and smallpox have similar signs and symptoms? *

Mark only one oval.

- Yes
- No
- I don’t know

48. Are flu-like symptoms (fever, chills, cough, runny nose, fatigue, headache, and muscle and backache) early signs or symptoms of human monkeypox (mpox)? *

Mark only one oval.

- Yes
- No
- I don't know

49. Are rashes (an area of irritated or swollen skin) one of the signs or symptoms of human monkeypox (mpox)? *

Mark only one oval.

- Yes
- No
- I don't know

50. Are vesicles (small, fluid-filled sacs) on the skin one of the signs or symptoms of human monkeypox (mpox)? *

Mark only one oval.

- Yes
- No
- I don't know

51. Are papules (tiny, raised bumps on the skin) one of the signs or symptoms of human monkeypox (mpox)? *

Mark only one oval.

- Yes
- No
- I don't know

52. Are pustules (bulging patches of skin filled with yellowish pus) one of the signs or symptoms of human monkeypox (mpox)? *

Mark only one oval.

- Yes
- No
- I don’t know

53. Is diarrhea one of the signs or symptoms of human monkeypox (mpox)? *

Mark only one oval.

- Yes
- No
- I don’t know

54. Is lymphadenopathy (swollen lymph nodes) a clinical sign that can help differentiate monkeypox (mpox) from smallpox? *

Mark only one oval.

- Yes
- No
- I don’t know

55. Can monkeypox (mpox) be prevented by properly cooking meat from potentially infected animals? *

Mark only one oval.

- Yes
- No
- I don’t know

56. Are hand sanitizers and face masks important for preventing monkeypox (mpox)? *

Mark only one oval.

- Yes
- No
- I don't know

57. Is paracetamol one of the management options for symptomatic monkeypox (mpox) patients? *

Mark only one oval.

- Yes
- No
- I don't know

58. Are antiviral drugs required in the management of human monkeypox (mpox) patients? *

Mark only one oval.

- Yes
- No
- I don't know

59. Are antibiotics required in the management of human monkeypox (mpox) patients? *

Mark only one oval.

- Yes
- No
- I don't know

60. Is there a specific vaccine for monkeypox (mpox)? *

Mark only one oval.

- Yes
- No
- I don’t know

61. Can the smallpox vaccine be used for the prevention of monkeypox (mpox)? *

Mark only one oval.

- Yes
- No
- I don’t know

62. Are people who received the chickenpox vaccine immunized against monkeypox (mpox)? *

Mark only one oval.

- Yes
- No
- I don’t know

63. Is there a specific treatment for monkeypox (mpox)? *

Mark only one oval.

- Yes
- No
- I don’t know

Attitude-Related Characteristics of Healthcare Workers Toward Prevention and Control of the Re-emergence of Human Monkeypox Infection in the Central Ethiopia Region.

Hint: Attitude items use a Likert scale with the following options: 1 = Strongly Agree, 2 = Agree, 3 = Neutral, 4 = Disagree, and 5 = Strongly Disagree.

64. I am confident that the global community can control the spread of monkeypox worldwide. *

Mark only one oval.

	1	2	3	4	5	
Stro	◯	◯	◯	◯	◯	Strongly disagree

65. I am confident that the Ethiopian Ministry of Health and the local population can effectively control monkeypox within the country. *

Mark only one oval.

	1	2	3	4	5	
Stro	◯	◯	◯	◯	◯	Strongly disagree

66. I am interested in learning more about monkeypox disease. *

Mark only one oval.

	1	2	3	4	5	
Stro	◯	◯	◯	◯	◯	Strongly disagree

67. I feel anxious that monkeypox could become a worldwide pandemic.

Mark only one oval.

	1	2	3	4	5	
Stro	◯	◯	◯	◯	◯	Strongly disagree

68. I believe that monkeypox can place an additional burden on the healthcare systems of affected countries. *

Mark only one oval.

	1	2	3	4	5	
Stro	◯	◯	◯	◯	◯	Strongly dsiagree

69. I am worried that monkeypox could be transmitted to Ethiopia. *

Mark only one oval.

	1	2	3	4	5	
Stro	◯	◯	◯	◯	◯	Strongly disagree

70. I believe that mass media coverage of monkeypox can positively influence its prevention at the global level. *

Mark only one oval.

	1	2	3	4	5	
Stro	◯	◯	◯	◯	◯	Strongly disagree

71. I am interested in learning more about the epidemiology of newly emerging diseases. *

Mark only one oval.

	1	2	3	4	5	
Stro	◯	◯	◯	◯	◯	Strongly disagree

72. I am interested in learning more about travel medicine.

Mark only one oval.

	1	2	3	4	5	
Stro	◯	◯	◯	◯	◯	Strongly disagree

73. I believe it is risky to travel to countries experiencing a monkeypox outbreak. *

Mark only one oval.

	1	2	3	4	5	
Stro	◯	◯	◯	◯	◯	Strongly disagree

74. I believe that the current prevention and control measures for monkeypox are sufficient. *

Mark only one oval.

	1	2	3	4	5	
Stro	◯	◯	◯	◯	◯	Strongly disagree

75. I am confident that the spread of monkeypox infection can be effectively controlled at the global level. *

Mark only one oval.

	1	2	3	4	5	
Stro	◯	◯	◯	◯	◯	Strongly disagree

76. I am willing to avoid contact with animals that could potentially harbor the monkeypox virus. *

Mark only one oval.

	1	2	3	4	5	
Stro	◯	◯	◯	◯	◯	Strongly agree

77. I am willing to manage monkeypox-infected patients as a frontline healthcare provider. *

Mark only one oval.

	1	2	3	4	5	
Stro	◯	◯	◯	◯	◯	Strongly disagree

78. I am confident in my ability to maintain standard precautions to prevent the transmission of the monkeypox virus. *

Mark only one oval.

	1	2	3	4	5	
Stro	◯	◯	◯	◯	◯	Strongly disagree

79. Adequate information about the monkeypox virus is essential for healthcare workers. *

Mark only one oval.

	1	2	3	4	5	
Stro	◯	◯	◯	◯	◯	Strongly disagree

80. Travel to countries affected by monkeypox should be restricted to prevent the spread of the disease. *

Mark only one oval.

	1	2	3	4	5	
Stro	◯	◯	◯	◯	◯	Strongly disagree

81. If I become infected with monkeypox, I will follow medical advice and adhere to isolation guidelines. *

Mark only one oval.

	1	2	3	4	5	
Stro	◯	◯	◯	◯	◯	Strongly agree

82. I believe that proper health education and safe patient handling are crucial to prevent monkeypox virus transmission between patients and between patients and healthcare workers. *

Mark only one oval.

	1	2	3	4	5	
Stro	◯	◯	◯	◯	◯	Strongly disagree

83. I believe that proper counseling by health workers to patients and attendants can help reduce the prevalence of viral diseases like monkeypox. *

Mark only one oval.

	1	2	3	4	5	
Stro	◯	◯	◯	◯	◯	Strongly disagree

84. I would like to receive training on monkeypox before any new cases are detected in our country. *

Mark only one oval.

	1	2	3	4	5	
Stro	◯	◯	◯	◯	◯	Strongly disagree

85. I am willing to take the monkeypox vaccine if it becomes available. *

Mark only one oval.

	1	2	3	4	5	
Stro	◯	◯	◯	◯	◯	Strongly disagree

86. Healthcare workers should be tested when they are in contact with someone infected.

Mark only one oval.

	1	2	3	4	5	
Stro	◯	◯	◯	◯	◯	Strongly disagree

87. I am willing to visit family members or friends who are infected with monkeypox. *

Mark only one oval.

	1	2	3	4	5	
Stro	◯	◯	◯	◯	◯	Strongly disagree

88. I believe I should practice more hygienic preventive measures because of the risk of monkeypox.

Mark only one oval.

	1	2	3	4	5	
Stro	◯	◯	◯	◯	◯	Strongly disagree

89. I believe that all individuals with a skin rash should be tested for monkeypox. *

Mark only one oval.

	1	2	3	4	5	
Stro	◯	◯	◯	◯	◯	Strongly disagree

90. I am concerned that monkeypox could become a new pandemic with an impact similar to COVID-19. *

Mark only one oval.

	1	2	3	4	5	
Stro	◯	◯	◯	◯	◯	Strongly disagree

91. I am interested in learning more about monkeypox.

Mark only one oval.

	1	2	3	4	5	
Stro	◯	◯	◯	◯	◯	Strongly disagree

92. I am interested in learning more about the epidemiology of newly emerging diseases. *

Mark only one oval.

	1	2	3	4	5	
Stro	◯	◯	◯	◯	◯	Strongly disagree

This content is neither created nor endorsed by Google.

Google Forms
